# Co-Creating a Digital Life-Integrated Self-Assessment for Older Adults: User Experience Study

**DOI:** 10.2196/46738

**Published:** 2023-09-26

**Authors:** Melissa J Böttinger, Elena Litz, Katharina Gordt-Oesterwind, Carl-Philipp Jansen, Nicole Memmer, Christian Mychajliw, Leon Radeck, Jürgen M Bauer, Clemens Becker

**Affiliations:** 1 Digital Unit, Center for Geriatric Medicine Heidelberg University Hospital Heidelberg Germany; 2 Network Aging Research Heidelberg University Heidelberg Germany; 3 Institute of Sports and Sports Sciences Heidelberg University Heidelberg Germany; 4 Department of Clinical Gerontology and Geriatric Rehabilitation Robert Bosch Hospital Stuttgart Germany; 5 Geriatric Center University Hospital of Tübingen Tübingen Germany; 6 Department of Psychiatry and Psychotherapy University Hospital of Tübingen Tübingen Germany; 7 Institute for Computer Science Heidelberg University Heidelberg Germany

**Keywords:** aged, self-assessment, mobile apps, mobile health, mHealth, community-based participatory research, co-creation, comprehensive geriatric assessment, mobile phone

## Abstract

**Background:**

Older adults are at increased risk of developing health disorders and functional decline. However, owing to time constraints and considerable effort, physicians rarely conduct comprehensive assessments to detect early signs of negative trajectories. If designed properly, digital technologies could identify health risks already at a preclinical stage, thereby facilitating preventive efforts and targeted intervention. For this purpose, a Life-integrated Self-Assessment (LiSA) tablet system will be developed through a structured co-creation process.

**Objective:**

This study aims to investigate older adults’ perceptions of different self-assessment domains, components affecting user experience, risks and benefits associated with LiSA, characteristics of potential LiSA users, and the LiSA concept in general.

**Methods:**

A total of 10 community-dwelling older adults aged ≥70 years were recruited. In total, 6 co-creation workshops were held and started with expert input followed by semistructured discussion rounds. Participants performed hands-on activities with a tablet, including testing of preinstalled self-assessment apps. All workshops were audio recorded and additionally documented by the researchers using flipcharts, notes, and photos. Qualitative content analysis was used to analyze the data following a deductive-inductive approach guided by the Optimized Honeycomb Model for user experience.

**Results:**

The group (mean age 77.8, SD 5.1 years) was heterogeneous in terms of previous technology experience and health status. The mean workshop duration was 2 hours (122.5, SD 4.43 min), and an average of 8 (SD 1.15) participants attended each workshop. A total of 11 thematic categories were identified, covering results on all research questions. Participants emphasized a strong interest in conducting a digital self-assessment of physical activity and function and sensory and cognitive functions and requested additional features such as recommendations for actions or reminders. LiSA was perceived as empowering and a motivator to engage in active health care planning as well as enabling shared and informed decision-making. Concerns and barriers included the lack of technical competence, feelings of frustration, and fear of being left alone, with negative assessment results. In essence, participants expressed a positive attitude toward using LiSA repeatedly and identified it as an option to increase the chances of maintaining independence when growing older.

**Conclusions:**

The co-creation participants supported the LiSA approach and were interested in performing regular self-assessments on a long-term basis. In their opinion, LiSA should include relevant assessments capturing physical activity and function and sensory and cognitive functions as well as recommendations for actions. It should be customizable to individual needs. These results will form the basis for a prototype. Iterative development and validation will aim to make LiSA accessible in the public domain as a reliable tablet-based system for self-assessment.

## Introduction

### Future Challenges for Health Care

By 2027, the old age–to–working age demographic ratio in Organisation for Economic Cooperation and Development countries will be almost 40%, and it will be >50% by 2050 [1]. The aging of the baby boomer generation will lead to health care needs that will most probably not be met by a shrinking workforce of health care professionals (HCPs) unless major changes are implemented in the health care system [2]. Older adults aged >70 years have an increased risk of developing health disorders. Approximately 30% to 40% will follow accelerated functional decline trajectories [3]. Cognition, mood, social contact, sensory function, and mobility often deteriorate gradually. In general practice offices, older patients often present with discrete functional impairments, which may hamper the early identification of risks [4]. To meet the challenges of an aging global population, health care delivery processes may benefit from digital technologies.

### Starting Points for Self-Assessment in Health

The World Health Organization *Integrated care for older people* guidelines stress the importance of health assessments and support for older adults’ self-management to prevent premature decline and foster healthy aging [2]. A comprehensive geriatric assessment (CGA) is a multidimensional process usually conducted by a multidisciplinary team (ie, physicians, nurses, social workers, and other HCPs) in hospitals, residential care homes, or community settings [5]. The current literature defines CGA as determining an older person’s medical, psychosocial, functional, and environmental risks and resources [6]. It goes beyond a disease-oriented medical assessment and allows for a more individualized and comprehensive care planning and follow-up [7]. Over the past 2 decades, numerous studies have shown that a CGA can benefit patients, society, and the health care system by identifying the early signs of negative trajectories [8-11]. Despite the positive effects of a CGA, there remains a *know-do* *gap* in most settings [11-13]. Implementation barriers include poor acceptance of preventive work [14], the lack of guidelines and professional interactions, and time and economic factors [12,13]. A promising solution to facilitate the scaled-up implementation of regular CGAs may be the integration of digital and patient self-service technologies into medical practice.

### Digital Technology and Older People

Owing to the COVID-19 pandemic and social isolation, the willingness of older people to use wireless information and communications technology (ICT) has further increased [15]. Smartphones are owned by >70% of people aged ≥70 years in the United States, and tablets are owned by almost 60% [15]. Nevertheless, the target group of older adults has specific usability requirements, and sovereign operation is particularly important for health apps to be used without supervision. Therefore, involving end users in the co-creation of health interventions is imperative. The literature now provides guidelines to support the usability of mobile health apps for older users [16], systematic reviews on factors influencing their acceptance of technologies are available [17], and tools such as those from the HEALTH CASCADE project for evidence-based co-creation of public health interventions [18] can be used.

### Current State of the Art in Digital Self-Assessment

In 2021, a total of 350,000 fitness, health, and medical apps were available for download in the Apple Store and Google Play Store [19]. This is comparable with approximately 160,000 in 2015, indicating a high interest in and demand for digital health apps. Compared with this enormous growth, the provision of apps as medical devices has been very slow owing to high authorization burdens [19]. Many countries are working to set up regulatory pathways [20]. Using activity trackers and wearables for heart rate, glucose, or oxygen saturation monitoring, citizens have started to collect their own health data, sometimes on a daily basis. However, currently, these data are often not factored in by HCPs. The development of digital self-assessment of cognitive, sensory, and physical functions is a rapidly developing process [21-27]. Recently, there have been some attempts using a comprehensive assessment approach [28,29]. The current landscape of digital health technologies reveals a market in which technologies are often developed commercially and rapidly but often at the expense of regulated medical product design, safety, and clinical validation [30].

### Proposing the Life-Integrated Self-Assessment to Address Problems and Potentials

We aimed to develop a *Life-integrated Self-Assessment* (LiSA) providing self-screening and monitoring for older adults to identify health risks early and facilitate efficient and targeted health care. LiSA is to be performed on a regular basis at home by people aged >70 years independently living at home. To our knowledge, this is the first approach toward a superordinate, tablet-based system for evidence-based, predictive self-assessments that provides users with individual, outcome-oriented, and scientifically sound recommendations for actions. LiSA’s development is designed as a process of co-creation, which we define as “an evidence-based methodology for the development, implementation and evaluation of innovations through continuous, open collaboration, interactional knowledge production and shared decision-making among key stakeholders, directed at improving public health” [31]. By using a co-creation approach, we aim to gain a deeper understanding of the target group’s needs regarding LiSA to develop a tailored and valuable solution with maximum user experience (UX). UX has become increasingly important in recent years. Research has focused on UX components that go beyond instrumental needs; include affective and emotional aspects of interaction; and understand the encounter with technology as subjective, contextual, dynamic, and complex [32].

In this study, we report the first step of the iterative LiSA co-creation process, which aimed to answer the following research questions (RQs):

1. RQ 1: Which assessments should or should not be part of LiSA?
2. RQ 2: Which components would affect the LiSA UX?
3. RQ 3: What benefits and risks do older adults expect regarding LiSA?
4. RQ 4: What characteristics might distinguish potential LiSA users from nonusers?
5. RQ 5: What do participants think about the LiSA concept in general?

## Methods

### Overview

In April 2022 and May 2022, we conducted a series of 6 workshops with older adults at the study center (Network Aging Research, University of Heidelberg, Germany). The co-creation process is described following the evidence-based co-creation guideline (PRODUCES+ [Problem, Objective, Design, [End-] Users, Co-creators, Evaluation, Scaling]) [33], which extends the previous PRODUCES framework [34]. The PRODUCES+ reporting checklist can be found in Multimedia Appendix 1 [33-41].

### Participant Recruitment

Convenience and stratified sampling methods were adopted to facilitate participant engagement [34]. We aimed to identify a diverse sample of older adults with sufficient heterogeneity in age, gender, health status, previous experience, and competence regarding ICT use. Participants who took part in a previous study [35] were contacted by mail. Those who agreed to participate were screened via phone. Inclusion criteria were age of ≥65 years, living at home, internet access at home, previous experience using ICT (eg, tablets, smartphones, or computers), and absence of acute or severe illnesses (eg, cardiac arrhythmia or planned surgery). Further exclusion criteria were subjective hearing or vision impairment leading to limitations in everyday life and inability to walk without assistive devices to ensure participants’ capability to fully participate in workshop content and discussions. To ensure accessibility to the study center in compliance with SARS-CoV-2 regulations, full vaccine protection was required. A total of 10 participants were included and provided informed consent to take part in the study.

### Co-Creation Workshops

#### Overview

The workshops were conducted by an interdisciplinary research team consisting of a geriatrician (CB), 2 physiotherapists (MJB and KG-O), a sports scientist (C-PJ), 2 psychologists (EL and CM), a sociologist (NM), a software engineer (LR), and an optometrist (MV). MJB moderated the workshops and was accompanied by 2 to 3 other members of the team, who contributed by giving short expert presentations, taking notes, and being available to support and answer questions during individual and group work. A brief description of the content of each workshop is provided in the following sections. Multimedia Appendix 2 contains a more detailed content and material description of the workshops.

#### Workshop 1

**Figure 1:**
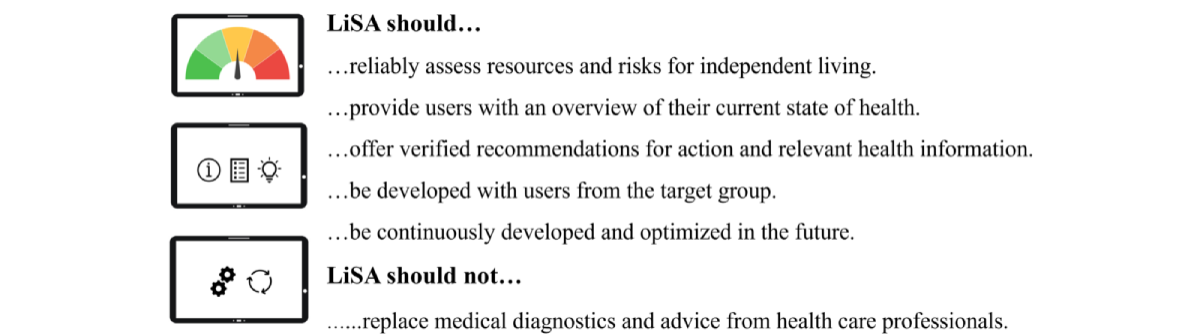
Life-integrated Self-Assessment (LiSA) concept presented to the participants.

Workshop 1 started with a round of introductions and information on the background and the concept of LiSA as well as the aims and agendas of the workshop sessions (Figure 1 and Multimedia Appendix 2) to ensure transparency with the participants [34]. To explain the term *digital self-assessment*, an example app for a fall risk self-assessment was shown to the participants (Table 1). A joint understanding of roles in the co-creation process was discussed in plenary to ensure that all co-creators had equal status within the group and responsibility to contribute their ideas [33]. After participants’ agreement on the agenda and roles was obtained, workshop 1 continued with participants sharing their first thoughts about the LiSA idea in plenary (RQ 5). In the next step, the card sorting technique was used to categorize and prioritize possible self-assessment contents (RQ 1). At the end of workshop 1, all participants were handed a tablet (Lenovo Tab M10 FHD Plus) and given the homework to test an app (Table 1), which should invite participants to be introduced to the basic tablet functions.

**Table 1:** Self-assessment apps and materials used during the workshops.

Number	Type of material and name (translation)	Goal	Home page; download; and scientific publication
1	Smartphone app: Aachener Sturzpass (fall risk prediction tool)	Fall risk self-assessment	Home page [42]; publication [43]
2	Tablet and smartphone app: starthilfe digital (digital starter kit)	Introduction to basic tablet functions	Home page [44]; download from the Google Play Store [45]; download from the Apple App Store [46]
3	Smartphone app: Up &Go	Instrumented Timed Up and Go test—measure functional mobility	We used a previous version of the app; download of the current version from the Google Play Store [47]; publication [27,48]
4	Print brochure: Bewegungspass (mobility passport)	Self-assess fitness and exercise	Download [49]; balance and mobility self-assessment based on the studies by Guralnik et al [50], Rikli and Jones [51], Howe et al [52], Berg [53], Tinetti et al [54], and Clemson et al [55]
5	Tablet app: TUCAN^a^	Cognitive assessment	Home page [56]; publication [23]
6	Print brochure: LUCAS^b^ Navigator	Self-test of functional competence	Download [57]; publication [58]
7	Web-based application: ZEISS Online Vision Screening	Self-assessment of visual function	Home page [59]
8	Tablet and smartphone app: Mimi Hearing Test	Self-assessment of hearing function	Home page [60]; download from the Google Play Store [61]; download from the Apple App Store [62]
9	Tablet app: smartVERNETZT (PRISM^c^)	Reduce social isolation and loneliness	Home page [63]; we used the currently developed German version of the PRISM system [64]
10	Tablet app: KOKU^d^	Home-based strength and balance exercise	Home page [65]; download from the Google Play Store [66]; download from the Apple App Store [67]; we used the German version of the KOKU app [68]

^a^TUCAN: Tuebingen Cognitive Assessment for Neuropsychiatric Disorders.

^b^LUCAS: Longitudinal Urban Cohort Aging Study.

^c^PRISM: Personal Reminder Information and Social Management.

^d^KOKU: Keep On Keep Up.

#### Workshops 2 to 5

Workshops 2 to 5 followed a similar agenda. After a short wrap-up of the last workshop, participants were invited to share their experiences with the homework. Each workshop contained a short expert presentation on the main topic of each workshop: *physical activity* (workshop 2; C-PJ), *physical function* (workshop 2; C-PJ), *cognition* (workshop 3; CM), *vision* (workshop 4; CB), *hearing* (workshop 4; MV), and *social and contextual factors* (workshop 5; NM). Each presentation was followed by participants sharing their knowledge and experiences of each topic to create a common understanding of each domain. In the next step, participants tested selected self-assessments, both analog and digital, in group or individual work mode (Table 1). This method was applied to stimulate participants’ thoughts about components that would affect the LiSA UX positively or negatively (RQ 2). Participants shared their experiences from these *try-out sessions* in plenary. The suitability of these self-assessments to be part of LiSA was also discussed (RQ 1). In workshop 5, a total of 2 apps currently under development [64,68] were presented to the participants to provide an outlook on possible follow-up interventions (Table 1). Between workshops, participants were asked to test further preselected self-assessments and complete questionnaires addressing their technology commitment and affinity. These are described in detail in the *Data Collection* section.

#### Workshop 6

In workshop 6, the card sorting of self-assessment domains (workshop 1; RQ 1) was repeated to ensure informed decision-making and gain information on participants’ awareness of the relevance of the suggested LiSA contents for the early identification of health risks. The differences between the card sorting results from workshops 1 and 6 were discussed in the group afterward. To stimulate discussion about user types (RQ 4), participants were presented with 4 fictional profiles representing older adults with different attitudes toward health and technology use (Multimedia Appendix 3). Working in tandems, participants were invited to become familiar with one persona and note their thoughts on whether and why this persona would be a LiSA user or nonuser. The results were shared and discussed in the group. The last part of workshop 6 focused on benefits and risks regarding LiSA (RQ 3). After a short expert input on data security (LR), participants discussed the benefits and risks they expected as a result of using LiSA. Workshop 6 concluded with participants sharing their thoughts about the LiSA idea in general (RQ 5) and feedback on the workshops.

### Data Collection

All workshops were audio recorded. During group discussions, researchers (MJB and EL) documented the discussion results on a flipchart and created a workshop protocol from an observer perspective. The group work processes and results were photographed. Participants provided written consent for the photos and audio recordings.

Before workshop 1, data on participant characteristics were collected using a paper-based questionnaire specifically designed for this study. The following data were obtained to verify the inclusion criteria and for sample description: sociodemographics (ie, age and gender), SARS-CoV-2 vaccination status, lifestyle (ie, living at home, living alone or not, former employment, participation in voluntary work, and physical activity level), health status (ie, hearing or vision impairment and acute or severe illnesses), and previous experience with technology use (ie, use of devices and fitness apps).

In total, 3 questionnaires on affinity and commitment toward technology and UX were used. The Affinity for Technology Interaction (ATI) scale [36] was handed out to the participants after workshop 1 to quantify their tendency to actively engage in technology interaction. The ATI has been demonstrated to be a reliable, valid, and economic tool for research applications, such as the characterization of user diversity. It contains 9 items rated on a 6-point Likert scale from 1 (*completely disagree*) to 6 (*completely agree*).

The Technology Commitment Short Scale [37] was filled out by the participants after workshop 2. It is a 12-item questionnaire using a 5-point Likert scale (1=*completely disagree*; 5=*completely agree*). It identifies 3 determinants of readiness to use technology: technology acceptance, technology competence, and technology control convictions. The Technology Commitment Short Scale has been developed to study the use of new technologies in older age for both research and practice and has good psychometric properties.

The short version of the User Experience Questionnaire (UEQ-S) [38] was filled out by participants for each self-assessment app they tested in workshops 2 to 5. The UEQ-S is the 8-item short form of the original User Experience Questionnaire (UEQ) and measures the subjective impression of users regarding the UX of products. This questionnaire was chosen as it is available in Germany, and the underlying UEQ has shown sufficient reliability and good construct validity [69]. Each item of the UEQ-S consists of a pair of terms with opposite meanings and can be rated on a 7-point Likert scale. The UEQ-S contains 2 subscales with 4 items each: pragmatic quality (eg, complicated—easy) and hedonic quality (eg, boring—exciting), with a total value reflecting the overall UX.

### Data Analysis

Quantitative data were analyzed descriptively using SPSS Statistics (version 27.0.1.0; IBM Corp). The mean, SD, minimum, maximum, median, range, and Cronbach α values were calculated. UEQ data were analyzed using the UEQ Data Analysis Tool (UEQ Team) [38,69]. MAXQDA Plus 2022 (version 22.3.0; VERBI GmbH) was used for verbatim transcription of the workshop audio recordings and qualitative content analysis [39] to answer RQs 1 to 5.

**Figure 2:**
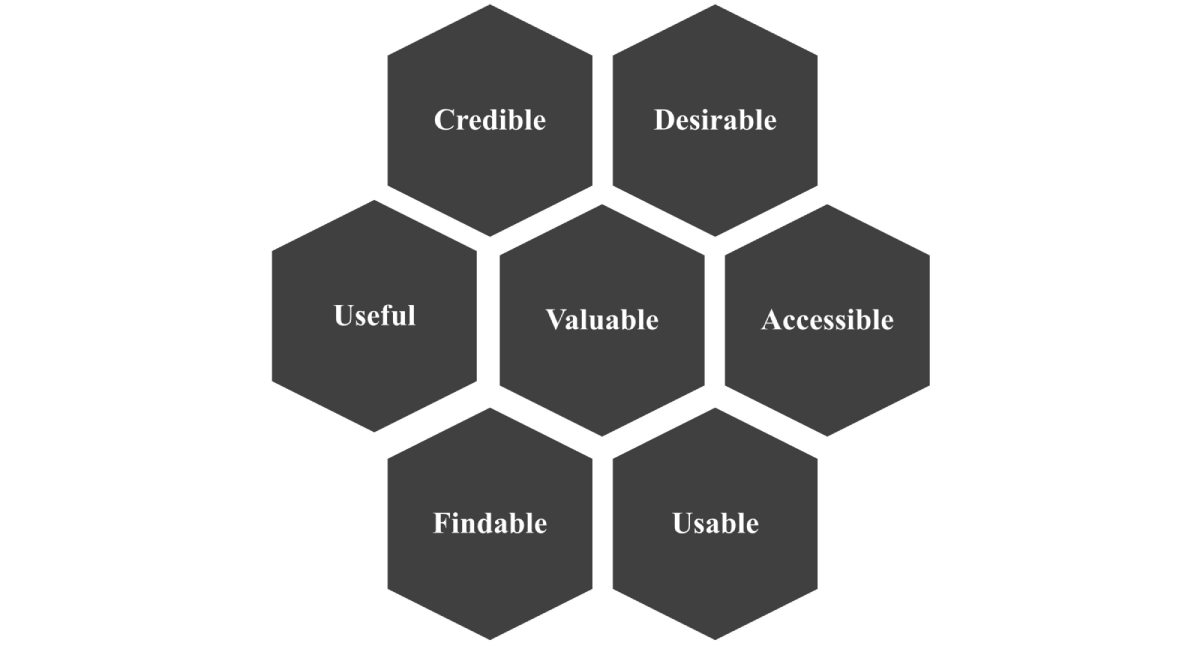
The Optimized Honeycomb Model for user experience (adapted from Karagianni [40], with permission from Katerina Karagianni and Morville [41], with permission from Peter Morville.)

For qualitative data analysis, the Optimized Honeycomb Model for UX [40,41] (Figure 2) served as a basic structure to categorize the components affecting the LiSA UX thematically (RQ 2). This model has been successfully applied in other UX studies in health research [70] and in a recent co-creation study aimed at improving the UX of a self-test app to assess balance function [26].

Before starting the data analysis, researchers (MJB and CB) agreed on the definitions of each Honeycomb Model category, oriented toward the original description [40,41] (Textbox 1).

**Agreed upon definitions for each Honeycomb Model category.**

- Usable: *What does the Life-integrated Self-Assessment (LiSA) need to be used with effectiveness, efficiency, and satisfaction?*
- Useful: *Which LiSA functions would be helpful to meet user needs?*
- Findable: *How does LiSA have to be set up and structured so that people can navigate it easily?*
- Credible: *What must LiSA be like to be safe, credible, and trustworthy?*
- Accessible: *How can access to LiSA be enabled?*
- Desirable: *What components could make LiSA emotionally attractive?*
- Valuable: *What higher goal and benefits should LiSA fulfill?*

The subsequent process followed a deductive-inductive approach to content analysis [39,71,72]: (1) reading and understanding all transcripts; (2) identifying meaning units according to RQs 1 to 5; (3) deductively sorting the meaning units into the 7 Honeycomb Model categories (findable, accessible, usable, desirable, credible, useful, and valuable); (4) inductively creating further categories and allocating meaning units to these categories regarding RQ 1, RQ 3, RQ 4, and RQ 5; (5) creating subcategories within all categories; and (6) viewing and assigning data from flipcharts, photos, and researchers’ notes to the categories.

### Ethics Approval

This study was approved by the University of Heidelberg Medical Faculty ethical committee (S-110/2022).

## Results

### Participant Characteristics

A total of 10 community-dwelling older adults aged between 68 and 85 years (n=6, 60% female and n=4, 40% male; mean age 77.8, SD 5.1 years) with previous experience using ICT and access to the internet at home were included. A total of 60% (6/10) of the participants lived alone, and 60% (6/10) of the participants had spent more than 2 and a half hours per week doing moderate or vigorous physical activities (eg, brisk walking) during the last 3 months. All participants were retired and formerly employed in tourism (1/10, 10%), fashion (1/10, 10%), banking law (1/10, 10%), health care (1/10, 10%), public service (1/10, 10%), armed forces (1/10, 10%), and the education system (4/10, 40%), suggesting a high level of education among the participants. In total, 20% (2/10) of the participants regularly engaged in volunteer work. None of the participants reported any acute or severe illnesses or subjective visual impairment; 40% (4/10) reported perceived hearing limitations. Participants had previous experience using a computer (10/10, 100%), smartphone (9/10, 90%), tablet (4/10, 40%), and smartwatch (2/10, 20%). In total, 90% (9/10) reported knowing how to open and send messages (eg, email) and search for information on the internet. A total of 20% (2/10) used fitness tracking apps.

The group’s mean ATI score over the 9 items was 3.20 (SD 0.84; Multimedia Appendix 4 [36,37]), indicating that the group had neither a very high nor a very low tendency to actively engage in intensive technology interaction. The wide distribution of values on the 6-point Likert scale shows diversity regarding affinity for technology within the group.

Mean scores on the Technology Commitment Short Scale were 3.22 (SD 0.43) over all 12 items, 2.89 (SD 0.89) for technology acceptance, 3.44 (SD 0.74) for technology competence, and 3.33 (SD 0.55) for technology control convictions (Multimedia Appendix 4 [36,37]), showing that the group’s readiness for technology was moderate. The range of values on the 5-point Likert scale indicated diversity regarding the participants’ readiness to use technology.

The UEQ-S was filled out by participants for each app they tried during the workshops. The results of the UEQ-S showed positive overall UX evaluations as well as high pragmatic quality scores for 86% (6/7) of the tested apps. Hedonic quality was rated positively for all the apps. The hearing test app yielded neutral evaluations regarding pragmatic quality and overall score. Multimedia Appendix 5 [38] shows in detail how the apps were rated on the UEQ-S.

Owing to vacation and illness, an average of 8 (SD 1.15) people were present at each workshop. A total of 10% (1/10) of the participants were excluded from the study after workshop 1 because of noncompliance with the workshop ground rules. A substitute participant was recruited, who then took part in workshops 3 to 6. The duration of the workshops ranged from 117 to 130 (mean 122.5, SD 4.43) minutes.

### Co-Creation Results for RQs 1 to 5

#### Overview

**Figure 3:**
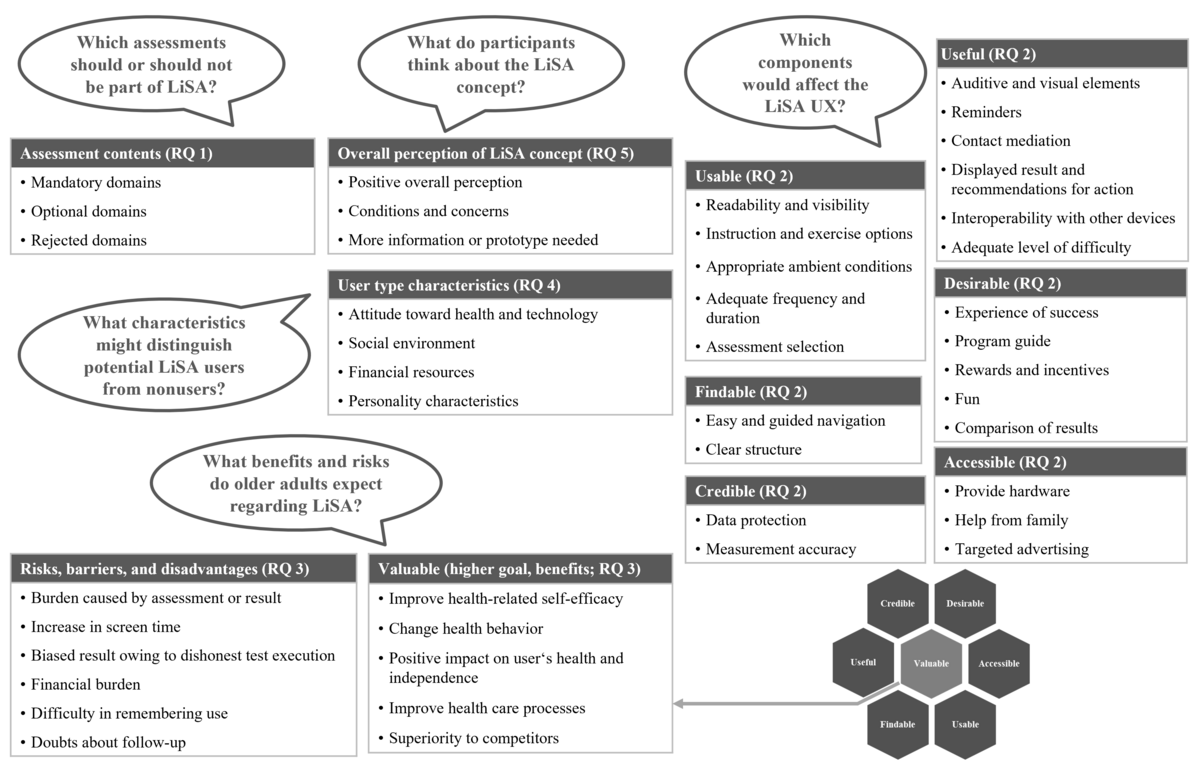
Categories and subcategories created to answer research questions (RQs) 1 to 5. LiSA: Life-integrated Self-Assessment; UX: user experience.

The deductive-inductive data analysis resulted in 11 thematic categories covering results on all RQs. The category *assessment contents* was inductively generated to answer RQ 1. In total, 6 of 7 Honeycomb categories—*findable*, *accessible*, *usable*, *desirable, credible*, and *useful*—were deductively created to address RQ 2. The seventh Honeycomb category, *valuable*, targets RQ 3 together with the inductively created category *risks, barriers, and disadvantages*. The categories *user type characteristics* and *overall perception of LiSA concept* were also inductively generated to answer RQ 4 and RQ 5, respectively. Within these 11 categories, 44 subcategories were created, as shown in Figure 3.

#### RQ 1: Which Assessments Should or Should Not Be Part of LiSA?

##### Overview

Participants’ opinions on self-assessment contents within LiSA were obtained in workshops 1 and 6 by asking them to sort prepared cards with standard CGA domains [5] and further assessments (eg, physical activity) into columns depending on whether they should be included in LiSA or not (Textbox 2). The results of the group work in workshop 6 were assigned to 3 subcategories within the category *assessment contents* during qualitative content analysis and will be described in the following sections.

**Life-integrated Self-Assessment contents—results from card sorting technique in workshop 6.**

**Mandatory domains**

- Physical activity (eg, steps/d)
- Physical function (eg, strength, balance, and fall risk)
- Cognitive function (eg, memory)
- Sensory function (eg, hearing and vision)

**Optional domains**

- Vaccination status
- Medication
- Comorbidities
- Home environment
- Affective function (eg, mood and depression)
- Nutrition
- Social environment (eg, loneliness)
- Pain
- Sleep

**Rejected domains**

- Incontinence
- Sexuality
- Financial situation

##### Mandatory Domains

The physical activity and cognitive and sensory function domains were perceived as necessary and important components of LiSA in workshops 1 and 6. Physical function (eg, strength) was not desired to be part of LiSA in workshop 1; however, participants considered physical function to be an essential part of LiSA in workshop 6 as they then discovered the strong connection between physical muscle strength and health:

Probably because the connection with health [and muscle strength] was not there. When you think of strength, you think of the gym. And that’s not the primary topic when you’re old and sick.

75 years; workshop 6

##### Optional Domains

When discussing the topics of vaccination, medications, and comorbidities, participants were unsure of whether this would be out of scope. They suggested capturing these domains in LiSA at the beginning. LiSA should then regularly remind people to attend medical checkups, but it should not include medical assessments:

But that’s a huge field when you’re sitting there in front of it, and you have to fill it all out. Will that be too much?

73 years; workshop 1

Assessments regarding home environment, nutrition, and affective function were initially rather unfamiliar or unknown to the participants in workshop 1. Then, in workshop 6, they emphasized that it would be important to assess and monitor these aspects, especially as they are often not considered in a physician’s visit. However, they were unsure of whether this would be feasible and appropriate as a self-assessment. Social environment, pain, and sleep were also seen as important aspects, but participants were not sure what a self-assessment in these domains would yield:

But, that there is the possibility [occupational therapist analyzing the home environment] that someone comes and looks at it, that makes sense. I would never have thought of that.

77 years; workshop 5

When it comes to financial status, what should the poor doctor say? Or nutrition. But there are also quite other things in the social environment, like contact, loneliness, and we know that the people are sicker there. And all this does not take place there at all.

73 years; workshop 1

I wonder what the app can do there, regarding loneliness.

77 years; workshop 6

##### Rejected Domains

Regarding incontinence, sexuality, and financial status, participants agreed in both workshop 1 and 6 that these domains should not be part of LiSA as they thought that people would not want to disclose information on these topics:

Of course, there are data that no one wants to give.

75 years; workshop 6

#### RQ 2: Which Components Would Affect the LiSA UX?

##### Overview

The results on RQ 2 were obtained both from aspects derived from other apps tested during the workshops and from participants’ own suggestions and ideas about the LiSA UX components. Multimedia Appendix 5 shows in detail the *dos and don’ts* derived from each app. All results on components affecting the LiSA UX were assigned to the following 6 Honeycomb categories and 23 subcategories.

##### Usable: What Does LiSA Need to Be Used With Effectiveness, Efficiency, and Satisfaction?

*Readability and Visibility*: This subcategory captures the participants’ needs for large font sizes and images as well as good visibility of app contents:

I would have preferred the symbols to be a bit larger because I had difficulty looking at them.

78 years; workshop 3

*Instruction and Exercise Options*: Participants desired to be introduced to the handling of the program as part of LiSA and stated that they would need to practice to successfully complete the self-assessments:

I thought it needs to be well explained. But I personally need more time to practice.

81 years; workshop 4

*Appropriate Ambient Conditions*: The need for appropriate lighting and a noise-free environment to perform the self-assessments was mapped to this subcategory:

I was very irritated by the conversations. A quiet place is certainly appropriate.

77 years; workshop 4

*Adequate Frequency and Duration*: Participants expressed different time specifications, ranging from 15 to 60 minutes per session and from daily to twice a year. At the same time, the participants noted that the appropriate frequencies can vary according to the content of the assessments:

No, you don’t do a hearing test every day. It’s different with physical exercises, which you do more often. You have to take that into account.

77 years; workshop 6

*Assessment Selection*: Participants had different opinions on whether the selection of assessments should be predetermined or self-decided. Some preferred to be flexible regarding when to perform which assessment and wanted the opportunity to decide that for themselves, as well as to avoid having to repeat assessments that had been recently completed with their HCPs. Others wanted LiSA to provide clear guidance on assessment performance:

You could make a selection at the beginning, where you say I’d like to try these areas now.

81 years; workshop 6

But also, not to have the feeling what I do today with LiSA that is up to me alone. So based on a certain randomness, but that LiSA then takes me by the hand a bit and says, so, this and that is on the agenda today.

68 years; workshop 6

##### Useful: What LiSA Functions Would Be Helpful to Meet User Needs?

*Auditive and Visual Elements*: Participants suggested integrating auditive and visual elements such as instructional videos, a voice assistant, or a reminder sound:

Via an acoustic signal. And maybe add a voice assistant to the whole app.

78 years; workshop 6

*Reminders*: A reminder function for the use of LiSA and for other appointments (eg, preventive care or HCP appointments) was desired by participants:

Maybe it should always remind you. That there is a pling sound and then LiSA is active and that means for the user, ah now I have to open it again and look.

78 years; workshop 6

And then, at the appropriate time, the app reminds you when which preventive checkup is due again.

75 years; workshop 1

*Contact Mediation*: Participants wished for LiSA to provide contact with technology counseling (eg, via senior citizen meetings) as well as with medical counseling (eg, physicians) to make appointments. There was also interest in a LiSA hotline and in having the possibility to share experiences and contact other users:

Such a hotline sign where you can inquire under a certain number. If you don’t know what to do, I think that would also be necessary. A direct one, without a waiting loop.

81 years; workshop 3

To get an idea of what helped others. When you’re in such a difficult situation and can’t see beyond it, then you do get a suggestion.

77 years; workshop 5

*Displayed Results and Recommendations for Action*: Participants wished to see not only the results after the assessment but also individual recommendations for actions to support daily health-conscious life:

I think what also plays a role, is that you get suggestions afterwards what you should do. I think that’s a very difficult step, because sometimes you know that there’s something wrong. And what should you do then? To find a solution.

73 years; workshop 2

*Interoperability With Other Devices*: Participants liked the idea of being able to connect LiSA with already available devices (eg, smartwatches), but they also feared increased complexity:

It would be nice [to use available devices]. Yes, but maybe that will make it too complicated.

78 years; workshop 2

*Adequate Level of Difficulty*: Participants agreed that the assessment tasks should not be too easy, and they expressed the idea of offering different levels:

But if it’s too easy, it’s not a test. Then you don’t see where you might have weaknesses. If I’m great everywhere, then I don’t need that.

80 years; workshop 4

##### Desirable: What Components Could Make LiSA Emotionally Attractive?

*Experience of Success*: Participants found that LiSA should be designed in such a way that users can experience a sense of achievement, for example, through the independent installation and operation of LiSA or the possibility to improve on assessment results over time. From the participants’ point of view, an experience of success feels motivating, encourages ambition, and causes a sense of pride. In contrast, being overchallenged by technology or assessments that are too difficult would lead to frustration and anger:

And then I’m really proud when I can say the next day, wow, it worked today. And that motivates me to try and do it again and again.

80 years; workshop 6

*Program Guide*: Participants liked the idea of an entertaining guiding element such as an animated character or avatar. They suggested that real people could act as ambassadors and program guides within LiSA. Participants were ambivalent about the extent to which this program guide should or should not be more senior oriented:

I am actually very happy when it is presented very senior-like, very detailed.

81 years; workshop 2

What I didn’t like was the speech, the way it was addressed. That was too senior-like for me.

78 years; workshop 2

*Rewards and Incentives*: This subcategory includes participants’ ideas on nonmaterial and material rewarding elements and incentives within LiSA. For example, insurance companies could provide a tablet device for free under the condition of using LiSA on it. In addition, rewards such as a point system or vouchers were suggested to increase adherence:

Maybe a reward system at the end of the week. That counts the number of points and then says here you have now reached 100 points. See if you can get 200 next week. A little incentive.

78 years; workshop 5

*Fun*: Participants wanted LiSA to be enjoyable as having fun with it would increase adherence and help them remember to use it:

For me, it would make sense in the first place if I enjoyed it. Then I do it voluntarily, then no one needs to remind me. The tests simply must be interesting and fun.

78 years; workshop 3

*Comparison of Results*: The participants had different opinions on the possibility of comparing results with those of other users. On the one hand, it could be competitive, fun, and stimulating. In contrast, it could also feel discouraging. Therefore, participants suggested that the possibility to compare results with those of others should be offered as an optional component:

I think it would drag me down. To be the worst of all the others I think I would give up.

80 years; workshop 3

##### Accessible: How Can Access to LiSA Be Enabled?

*Provide Hardware*: Participants had different ideas on how to access the LiSA tablet. They were in disagreement on whether the users themselves should bear the costs. It was suggested that insurance companies could offer the tablets to their customers:

It must be provided, only then it will work out.

78 years; workshop 6

And if you need a tablet to use it, you have to say, ok I’ll go for it and buy a tablet.

80 years; workshop 6

*Help From Family*: Being supported by family is an important factor in the use of LiSA. Most participants had received help from family members in the past in dealing with technology. However, they were unsure of the extent to which families could also help with a new program such as LiSA. Furthermore, there was a desire to be able to use technology successfully on their own in the future:

I actually want to be able to do it on my own.

81 years; workshop 3

*Targeted Advertising*: Participants felt that it was difficult to develop a *one-size-fits-all* approach. To enable widespread access to LiSA, participants believed that target group–specific advertising and communication are needed. They mentioned senior centers, television, and word-of-mouth recommendations as possible advertising channels, especially for older adults without internet access. It was suggested that different LiSA versions be gradually developed and offered to better address the different target groups:

There are many different people who will use it. And I think you can’t develop something optimal for everyone.

80 years; workshop 5

It would also be possible to set up LiSA courses in senior centres.

77 years; workshop 4

##### Credible: What Must LiSA Be Like to Be Safe, Credible, and Trustworthy?

*Data Protection*: Participants expressed a lack of knowledge on this topic. From their viewpoint, data protection was an illusion, and one simply must accept that there is no absolute guarantee of security. They had different opinions on the consequences that might occur if LiSA were to collect and share data with HCPs or other third parties. They did not want the data to be sold to insurance companies. From the participants’ point of view, there should be education in LiSA about data protection to provide transparency and overcome possible concerns:

Either you are not on the internet at all, or you have some risks.

78 years; workshop 6

*Measurement Accuracy*: The participants had a critical attitude toward the objectivity of the self-report assessment. Regarding mood or loneliness, they considered the comparison between self- and external assessment by another person to be helpful. In the case of the hearing and vision tests, the participants found it convincing and face-valid if the tests were structured similarly to those used by HCPs and if the goal behind the tasks within the assessment was recognizable for users. Participants’ thoughts on reliability and validity included that LiSA measurements must consider the placement of the device, the time of day, and the individual form on the day:

Or maybe I would ask other people. How do you feel about me? Because some say, listen, you’re always just sitting there in your apartment. And you’re totally happy about it. And the others say, no and that’s really bad and that’s already depression.

78 years; workshop 5

If I do this three times a day, and then the result is not the same.

68 years; workshop 4

##### Findable: How Does LiSA Have to Be Set Up and Structured So That People Can Navigate It Easily?

*Easy and Guided Navigation*: Clearly labeled buttons would help participants navigate and be guided within LiSA. Furthermore, participants liked the possibility to continue where they left off. Assessments that have already been completed should be deactivated within LiSA:

That should be easy, that you click on Done or Back or so. That you also find back.

73 years; workshop 3

I would like that the tests that I have already done are no longer accessible. I don’t want to do it twice and get different results. That would then have to be locked.

78 years; workshop 4

*Clear Structure*: Participants had several ideas on how to provide a clear higher-level structure by integrating different assessment domains into LiSA. A possibility would be to create links within LiSA that lead to other assessment apps or websites. However, participants were afraid that this could also be confusing. Another suggestion was to embed all individual assessments as modules in LiSA so that users would only need to use 1 program. Participants saw it as important that assessments be presented in a structured and clear overview:

It’s all interesting for sure, all the possibilities. But I think as an older person, clarity should have priority. If there is too much on offer and you can’t cope with it, then you don’t do it.

81 years; workshop 3

#### RQ 3: What Benefits and Risks Do Older Adults Expect Regarding LiSA?

##### Overview

The aspects from the workshops that relate to the higher goals, opportunities, and benefits of LiSA were assigned to the Honeycomb category *valuable* and thematically assigned to 5 subcategories. A separate category was created for the anticipated risks, barriers, and disadvantages that were expressed by participants. Within this category, 6 subcategories were created during qualitative content analysis.

##### Valuable: What Higher Goal and Benefits Should LiSA Fulfill?

*Improve Health-Related Self-Efficacy*: Participants perceived benefits of using LiSA to self-assess and monitor their own health status. They liked the idea that LiSA provided information on their health status and gave them a better self-estimation. They hoped to achieve more self-control regarding health decisions and appreciated the opportunity to perform the assessments in LiSA first instead of going straight to the physician:

A certain awareness of your estimation of yourself would not be wrong.

85 years; workshop 1

Well, I must say, I am someone who never goes to the doctor. Otherwise, I find something like that [LiSA] always better than going to the doctor. Because when I see, oh that was better last time, then I go to the doctor or somewhere.

81 years; workshop 4

*Change Health Behavior*: From the participants’ perspective, the displayed results and recommendations for action could help change their health behavior for the better:

So, I think that’s not bad if you get feedback on how bad you are, for example. Then I have to do something in that direction.

73 years; workshop 3

*Positive Impact on User’s Health and Independence*: This category includes participants’ reflections regarding the fact that LiSA could positively affect their own health and independence in old age:

This is really an interesting thing and I already think that this will help me in terms of health.

75 years; workshop 6

And this is where I see the benefit now. I want to be independent of the help from children for as long as possible.

81 years; workshop 6

*Improve Health Care Processes*: Participants stated that LiSA could empower users to prepare for physician visits, help physicians make diagnoses, and support patient education. They imagined that users would visit the physician earlier if necessary and would be more likely to attend preventive care appointments because of the LiSA results:

Something I can present that he [the doctor] can then review and get an idea that will help him make a diagnosis. A service that also forces the individual [doctor] to explain something. That is often missing in the medical examination. That would be a goal.

85 years; workshop 1

To say I’ll see what preventive checkups I need to attend. Did I think of everything? Preventive checkups at 70, preventive checkups at 80.

73 years; workshop 6

*Superiority to Competitors*: More generally, participants emphasized that the user should benefit from LiSA being superior to other digital offerings:

It must be better than what I already have.

78 years; workshop 6

##### Risks, Barriers, and Disadvantages: What Risks Do Older Adults Expect Regarding LiSA?

*Burden Caused by Assessment or Result*: The participants expressed concern that an overdemanding test procedure or a poor test result could cause a burden for users:

If you would score now very badly, and then you sit alone at home. I don’t know what to do then. I just imagined that there could also be a senior citizens’ meeting place and that there could be such a contact point.

73 years; workshop 3

*Increase in Screen Time*: The presumption that LiSA leads to more screen time was pointed out as a possible negative consequence:

The fact that you’re even more stuck to the screen is a disadvantage, of course.

77 years; workshop 6

*Biased Results Owing to Dishonest Test Execution*: It was mentioned that users would cheat on self-assessments to obtain a better result:

So, I don’t know if I would be so honest with myself if I were tested on that. I don’t know.

78 years; workshop 5

*Financial Burden*: If the data were passed on to insurance companies, LiSA could lead to financial disadvantages for every user from the participants’ perspective. The health care system could also be affected by additional costs if LiSA resulted in more medical consultations:

So, the insurance companies, they’re always mentioned there. That if you do that [LiSA], then the insurance company says, oh God, now he’s sick, he has to pay more for insurance.

73 years; workshop 6

Either we assume that someone who uses LiSA is already a health-conscious or illness-conscious person. And they will go to the doctor more often than others. So, they will cause more costs than someone who does not use LiSA.

75 years; workshop 6

*Difficulty in Remembering Use*: A possible barrier to long-term and regular LiSA use according to the participants could be forgetting to use LiSA:

My only concerns are that hopefully my memory will also allow me to remember to check something. For example, if it says daily or once a week, that I really remember it too.

80 years; workshop 1

*Doubts About Follow-Up*: Concerns were raised about the extent to which follow-up after LiSA could be ensured. Participants doubted that they could discuss LiSA outcomes with physicians or that it might take too long to obtain an appointment with an HCP after identifying emerging problems in LiSA. In addition, there are domains for which the ability of LiSA to make recommendations to improve is restricted, such as social contacts:

Taking that to the doctor, that doesn’t work at all, I assume that already. That doesn’t work.

75 years; workshop 6

I find it quite difficult, for example, with contacts. If someone has few social contacts, how does he change that?

80 years; workshop 5

#### RQ 4: What Characteristics Might Distinguish Potential LiSA Users From Nonusers?

##### Overview

The following results are derived from the findings of the tandem work with the personas in workshop 1 as well as the spontaneously expressed thoughts of the participants regarding possible LiSA users and nonusers during the other workshops. Multimedia Appendix 3 provides a description of the 4 personas and the results of the tandem work on why these personas would be users or nonusers. All results regarding RQ 4 were assigned to 4 subcategories within the category *user type characteristics* during qualitative content analysis.

##### Attitude Toward Health and Technology

The participants felt that it would be easier to reach people who are already motivated to care about their health, who already use a tablet, or who are interested in engaging with technology. Even though it was considered difficult, they found it crucial to reach people with little health motivation and technology competence so that these individuals, as well as the health care system, could benefit from the advantages:

I think Anita [persona with negative attitude toward health and technology], we would have to include her absolutely, because probably this group of people are the most expensive for the health care system.

78 years; workshop 6

##### Social Environment

Participants saw difficulties for users who could not expect support with technical issues from friends or family. However, these users should still be reached through training:

There you have to try to fix that [lack of skills] with training.

68 years; workshop 6

##### Financial Resources

As financing of the LiSA hardware was not predefined at the time of the workshops, the participants considered whether LiSA would then only be usable by people who owned a tablet or could afford to purchase one:

But if you have financial worries, you don’t use a LiSA app, I would say. Yes, the hardware must be there first.

78 years; workshop 6

##### Personality Characteristics

Another decisive criterion for LiSA use from the participants’ point of view was personality. They explained that some older people who are anxious or hesitant in general or have a change-averse personality would not use LiSA:

But there will certainly be those who say, I don’t need that, I have enough friends, I have way too much, I don’t know how I’m going to manage that with my schedule. That also exists. But they probably wouldn’t do that.

80 years; workshop 5

Sometimes people want to stay in their current state, they don’t want to be motivated. And in my experience, older people in particular don’t always want to hear, do this, from younger people, because they’re not in that situation. I think that people would like to stay more among themselves in the same generation.

81 years; workshop 6

#### RQ 5: What Do Participants Think About the LiSA Concept?

Participants’ overall perceptions and thoughts on the LiSA concept were summarized in the category *overall perception of LiSA concept* and were thematically assigned to 3 subcategories.

##### Positive Overall Perception

Participants were mostly positive and interested in using LiSA at home on a regular basis:

I find that interesting, I would certainly like to use that.

80 years; workshop 1

I hope that I still live to see the LiSA project and that it does not last too long, because I am one of the older ones and would actually like to use this for a few more years.

81 years; workshop 6

I think I could do this well on my own at home.

81 years; workshop 4

##### Conditions and Concerns

Overall conditions that must be met for LiSA to be used were expressed by participants and described in the results section for RQ 2 (UX) and RQ 3 (risks and benefits):

However, it would also have to be user-friendly.

80 years; workshop 6

So I’m open-minded, but at the same time I’m a little afraid of whether I’ll be able to cope with the whole thing when I’m on my own.

81 years; workshop 3

##### More Information or Prototype Needed

For some participants, it was too early to form an opinion on the LiSA concept as LiSA was not entirely predefined by the research team at the time of the workshops. Participants expressed the need for a prototype to better evaluate LiSA:

I still have no real idea what would be possible with the program.

68 years; workshop 1

I would have to try it and then try it again a few days later and then see the result.

78 years; workshop 5

## Discussion

### Principal Findings

The co-creation process was a mutual learning experience toward the development of LiSA. Overall, the participants in this co-creation process had a positive attitude toward the regular use of LiSA. The main expectations of the participants from LiSA were to collect valid and relevant data to have a better control of their health status, be better prepared for visits to their physicians, and be able to identify and respond earlier to risks. Their overall goal was to improve or maintain their own health status for as long as possible, thereby keeping their independence and autonomy.

Participants favored the ability to individually determine the frequency, duration, and scope of LiSA. Consistent with the recommendation of another study examining the usability of mobile health apps for older adults [16], LiSA should include a default selection of mandatory domains and the possibility to enable more functionality (ie, optional assessment domains). This would also be consistent with the personalized and iterative nature of a CGA [73]. The domains of physical activity and capacity as well as sensory and cognitive function were prioritized by the participants and, therefore, should be integrated into LiSA. However, it may be that participants prioritized these above all other assessment domains mainly because they already knew the assessments in these areas from their own experience or because they most likely noticed physical, sensory, or cognitive deterioration in their peer groups and were worried about becoming affected themselves. In contrast, participants may have rejected the domains of sexuality and incontinence out of embarrassment or because they believed that incontinence is a natural and inevitable consequence of aging [74]. Therefore, a balanced approach to user preference and medical expertise should be followed to define mandatory and optional assessments within LiSA. This will not only facilitate the integration of all relevant domains for risk identification into LiSA but also ensure a positive UX.

The participants highlighted that the identification of risk factors was relevant, but they expected problem-solving suggestions and a timely follow-up. LiSA should not be reduced or limited to an alarm function, leaving the participant alone with it. Another study on a self-test app to assess balance function showed similar results. Participants wanted not only to be notified when physical function was declining but also to receive guidance on how to exercise [26]. The provided information (eg, pointing out opportunities for social interaction, such as senior centers in the area) and recommendations for actions (eg, recommending consulting an appropriate expert or referring to a training intervention) within LiSA should be evidence-based and precise to ensure that they do not cause unnecessary medical visits. For people with physical or social access barriers to medical care (eg, remote rural areas), further digital interventions such as video consultation or training apps could also be offered as a follow-up to LiSA assessments.

The setting in which LiSA could be used was left open on purpose by the research team at the beginning of the workshops. The participants discussed that LiSA could be used solely as a private self-assessment or as a preclinical tool to prepare for a physician’s visit. The transfer of data to physicians (either electronically or independently brought along by the patient) has the potential to promote CGA implementation as reliable data would then already be available as a basis for further, specific assessments. However, the transfer of data to third parties such as physicians or health insurance companies was viewed with skepticism. In accordance with the participants’ opinions as well as a guideline to support mobile health app design for older users [16], LiSA should ensure transparency and users’ autonomy and control over their own data. Providing offline access would ensure data security and also avoid interruptions because of poor internet connectivity.

The level of technological competence as well as the type and number of available technical devices can vary greatly in the target group of older adults. Therefore, LiSA should allow for the interoperability of different devices such as smartphones, step counters, and tablets to exploit users’ existing individual resources and skills. Low technological competence and a negative attitude toward health and technology were the main characteristics of potential nonusers mentioned by participants. Other nonuser characteristics were a low level of social support, lack of financial resources, and anxious and change-averse personality traits. Workshop participants felt that no one should be excluded from the LiSA target group. However, realistically, there are criteria that may prevent the regular use of LiSA (eg, significant cognitive or visual impairment). To achieve accessibility and acceptance among different user types, personalized LiSA versions could be offered in perspective, such as a single-device version for people who own only one technical device. Possible further strategies to overcome user barriers are presented in Multimedia Appendix 3.

As in our group, there will be selective users in the population of older adults who like to compare apps and then select the best one. This means that development and subsequent maintenance must also consider the comparison with competitor apps to meet the disparate expectations and wishes of users. The integration of incentives into LiSA to increase attractiveness was proposed by participants but must be critically considered. Offering financial incentives such as vouchers or rebates might undermine intrinsic motivation and lead to a greater likelihood of disuse or manipulation of test results. However, cognitive evaluation theory predicts that, if such a reward is perceived as confirming an individual’s autonomy rather than controlling behavior, it would enhance intrinsic motivation [75].

### Strengths and Limitations of the Co-Creation Process

The main strength of this study lies in the high engagement of the participants, which is also shown by the high adherence rate and time they invested without any financial compensation. The 6 co-creation workshops were carried out as planned, and the atmosphere during the workshops was characterized by appreciation, trust, and constructivism. Participants engaged in the interaction with the research team as well as with the group, which is reflected in the depth and scope of the results. Different approaches were adopted throughout the co-creation process and reporting to strengthen the study’s validity and trustworthiness and increase the impact of the results [39]. During the group discussions, methods for securing results [34] were used to ensure a correct understanding of the participants’ comments. In addition, regular participant evaluations [33] were conducted to assess satisfaction with the co-creation process. The presentation of methods and results was guided by current guidelines [33,34]. Workshop contents and analysis procedures were described transparently to enable the comprehensibility of the methodological procedure. The use of quotations shows the connection between the data and the results, indicating the richness and diversity of the material. The UX Honeycomb Model proved to be an appropriate and helpful framework for categorizing our findings, and we recommend it for use in further UX research.

It should be noted that the results may have been biased by participants being similar in terms of ethnicity, cultural background, and high educational level. This makes it difficult to transfer the results to other contexts with more diversity. More multilayered data could have been obtained through the additional collection and analysis of video data, especially during small-group work. Another limitation of this study is that the qualitative content analysis was conducted by 1 person. Owing to the limited sample size and cultural setting, the results are not directly transferable to the general population of older adults.

### Future Perspectives

As suggested by the participants, the next step is to develop a LiSA prototype building on the findings from the co-creation process. From the participants’ perspective, this prototype is needed to thoroughly evaluate LiSA. In a follow-up study with the prototype, all components of UX (UX before, during, and after use) will be investigated further. The participants found that a one-size-fits-all approach should not be aimed for. As a possible first step into the consumer market, it might make sense to start with a version for users with few barriers. This version could then be iteratively tested, developed, and expanded to overcome barriers gradually and to be able to offer LiSA to a larger target population in the long term.

The following relevant stakeholders should be involved in future co-creation processes. Focus groups with general practitioners and other HCPs (eg, physiotherapists and optometrists) should be held to discuss readiness and potential barriers to integrating data collected in LiSA into appointments with HCPs. In addition, data privacy experts should be involved to ensure the security of user data within LiSA. Health insurance companies should also be considered as stakeholders to discuss possible funding opportunities, such as the provision of hardware to their policyholders. Finally, family members should also be involved to capture their perspective and needs to support older family members in their use of LiSA.

Further research steps will be the examination of the test quality criteria (ie, test-retest reliability and cross-validation) to verify whether LiSA provides comparable data with those of a standard CGA.

### Conclusions

We co-created the LiSA concept with 10 older adults, an approach toward LiSA to identify risks early and facilitate the targeted management of older adults’ health. The study design and chosen co-creation methods promoted an intensive discussion and differentiated insights into the ideas, expectations, and concerns of the target group. The co-creation participants supported the general concept and ascribed a high value and great interest to LiSA. The core assessments identified were physical activity and capacity and sensory and cognitive function. Customizable scope and content, as well as recommendations upon assessment results, were requested. On the basis of this study, a prototype will be designed, validated, and iteratively developed, including further co-creation processes with different stakeholders and including older adults with a lower educational level. Five take-home messages from this co-creation study are listed in Textbox 3.

**Take-home messages.**

- *Carry on*: the participants confirmed that the Life-integrated Self-Assessment would be relevant to them; further developments were recommended.
- *Repeat*: participants were interested in performing self-assessments on a regular basis and in the long term.
- *Less is more*: the scope should not be too extensive but focus on the most important assessments (physical activity and capacity as well as sensory and cognitive function).
- *Individualize*: content and scope should be customizable to the user’s needs.
- *Think ahead*: clear recommendations derived from assessment results were expected.

## Appendix

Multimedia Appendix 1 PRODUCES+ (Problem, Objective, Design, [End-] Users, Co-creators, Evaluation, Scaling) co-creation reporting checklist.

Multimedia Appendix 2 Overview of the workshop contents.

Multimedia Appendix 3 Description of personas, results from the tandem work, and strategies to overcome potential user barriers.

Multimedia Appendix 4 Results from the Affinity for Technology Interaction and Technology Commitment Scale.

Multimedia Appendix 5 Participants’ ratings of the tested apps on the short version of the User Experience Questionnaire and dos and don’ts regarding the Life-integrated Self-Assessment.

## Abbreviations

ATI: Affinity for Technology Interaction
CGA: comprehensive geriatric assessment
HCP: health care professional
ICT: information and communications technology
LiSA: Life-integrated Self-Assessment
PRODUCES+: Problem, Objective, Design, (End-) Users, Co-creators, Evaluation, Scaling
RQ: research question
UEQ: User Experience Questionnaire
UEQ-S: short version of the User Experience Questionnaire
UX: user experience
