# Supplementary material for: Co-Creating a Digital Life-Integrated Self-Assessment for Older Adults: User Experience Study
Source: JMIR Aging. 2023 Sep 26;6:e46738. doi: 10.2196/46738 (PMC10565622; doi:10.2196/46738)
Supplement: Multimedia Appendix 1 [file aging_v6i1e46738_app1.docx]

# Multimedia Appendix 1: Problem, Objective, Design, (End-) Users, Co-Creators, Evaluation, Scaling (PRODUCES+) co-creation reporting checklist.

| **Stage** | **Checklist Item** | **Response** |
| --- | --- | --- |
| **Planning** | | |
| How was the aim of the study framed? | Use each element of the PRODUCES framework (PRoblem, Objective, Design, (end-) Users, Co-creators, Evaluation and Scalability)  Which frameworks did you use? | Utilising Participatory Health Research (Design) to develop (Objective) and validate (Evaluation*), with academic researchers and older adults (Co-creators), a digital life-integrated self-assessment (LiSA) (Scalability) for early identification of risks to independent living (PRoblem) in community-dwelling older adults (end-Users).  *Evaluation/validation was not part of the present study. |
| Planning Methods | Which methods did you use? Why? | convenience/stratified sampling: to recruit a heterogenous sample (age, gender, health status, prior experience and competence regarding ICT use)  Formulation of qualitative research questions: to collect and explore opinions, experiences, needs, concerns and attitudes of potential end-users |
|  | Were there any methods you used that are not mentioned in this guideline? If yes, please list them: | - |
| Planning Frameworks | Which frameworks did you use? Why?  Were there any frameworks or models you used that are not mentioned in this guideline? If yes, please list them: | PRODUCES framework as a guideline to plan the co-creation process [33,34]  Optimized Honeycomb Model [40,41]: to prepare questions for the group discussions regarding possible UX components  SWOT technique to prepare questions for the group discussions regarding strengths, weaknesses, opportunities, and threats related to LiSA |
| Explain the sampling  procedure | Explain the criteria used for sampling: | convenience and stratified sampling:  We aimed to identify a diverse sample of older adults with sufficient heterogeneity in age, gender, health status, prior experience, and competence regarding ICT use. Participants who took part in a previous study [35] were contacted by mail. Those who agreed were screened via phone. Inclusion criteria were age ≥ 65 years, living at home, internet access at home, prior experience in using ICT (e.g., tablet, smartphone, computer) and absence of acute or severe illnesses (e.g., cardiac arrhythmia, planned surgery). Further exclusion criteria were major hearing or vision impairment and inability to walk without assistive devices. To ensure accessibility to the study centre in compliance with SARS-CoV-2 regulations, full vaccine protection was required. |
|  | In what settings did sampling occur? | community-dwelling older adults were recruited from a previous study |
|  | How many individuals engaged as co-creators (academic/non-academic stakeholders)? | 10 community-dwelling older adults, 9 researchers/stakeholders |
|  | Describe the co-creators (demographics/groups/  other characteristics of interest) | community-dwelling older adults: 6 females, 4 males, mean age = 77.8 ± 5.1), 6 were living alone, 6 were moderately/vigorously active. All participants were retired, 2 were regularly engaged in volunteer work. None of the participants had any subjective visual impairment; 4 reported perceived hearing limitations. All participants had access to the internet at home and prior experience in using ICT: computer (n=10), smartphone (n=9), tablet (n=4) and smart watches (n=2). Nine reported knowing how to open and send messages (e.g., E-Mail) and search information on the internet. Two used fitness tracking applications.  researchers/stakeholders: a geriatrician, two physiotherapists, a sports scientist, two psychologists, a sociologist, a software engineer, and an optometrist |
| **Conducting** | | |
| How was ownership  manifested? | Explain the methods used to manifest ownership (for example, branding the group, identifying the rights and responsibilities of the group): | WS1: group discussion about roles and responsibilities during the co-creation process to ensure all co-creators have the right and equal status within the group and their responsibility to contribute their ideas |
| Procedure Components | What level of participation was there from the co-creators? | Researchers and end-users strove to have equal participation. All co-creators contributed with their input to the discussion rounds. |
|  | How was the overall aim presented? | WS1 started with the information on the background, the aim and the concept of LiSA as well as the aims and agendas of the planned workshop sessions. |
|  | How was the purpose of each meeting presented? | Each workshop started with the presentation of its aim. The aim of the current workshop and an outlook on the goals for the next sessions were additionally displayed on a slide. |
|  | What were the rules and responsibilities of participation agreed upon? | Joint understanding of roles in the co-creation process, the right to equal status within the group and the responsibility to contribute their ideas were communicated to the participants and discussed in plenary. The following role tasks were formulated and agreed upon:  Participant role: report from your perspective, tell what you need and what you want, explain your point of view or the point of view of other people from your social environment, describe what you expect from digital technologies.  Researcher role: listen and ask questions, learn what users think and feel, collect all thoughts and ideas, develop technology that meets end-users’ desires and needs, provide background information when needed. |
| Conducting Methods | Which methods did you use? Why? | Optimized Honeycomb Model for UX [40,41]: to ask specific questions about the different aspects (honeycombs) of the Honeycomb Model in the discussion sessions.  SWOT technique to ask specific questions regarding strengths, weaknesses, opportunities, and threats related to LiSA  WS2-5: Field Testing of Prototypes/already existing self-assessment apps:  to provide participants with examples of what self-assessments could be like, to identify components affecting user experience, derive dos and don’ts for LiSA, tandem and individual work to have the opportunity to test technology for themselves and to observe others using it |
|  | Were there any methods you used that are not mentioned in this guideline? If yes, please list them: | WS1+WS6: Card sorting:  to initially provide ideas for assessment domains and to allow prioritization; adding new domains by the participants was also possible, card sorting was repeated in WS6 to identify possible changes in prioritization.  WS6: Personas (4 fictional persona profiles representing older adults with different attitudes towards health and technology use): to stimulate the discussion about user types, to collect characteristics that might distinguish LiSA users from non-users |
| Procedure Methods | Which areas did the co-creators require upskilling? | To explain the term “digital self-assessment”, an example app for a fall risk self-assessment was shown to the participants.  To familiarize themselves with the basic functions of the tablet, the participants were given a tablet and given the task of testing an application.  Researchers were upskilled regarding participants’ opinions, experiences, needs, concerns and attitudes towards LiSA. |
|  | What previous evidence was reviewed, and how? | WS1 started with the information on the background, the evidence on comprehensive geriatric assessments, and the concept of LiSA. Each workshop contained a short expert presentation on the main topic followed by participants sharing their knowledge and experiences on the respective topic in order to create a common understanding of each assessment domain. |
|  | If a prototype was developed, describe the prototype and the prototyping process: | - |
|  | Describe the frequency and duration of meetings: | 6 Workshops (1WS/week), the duration of the workshops ranged from 117 to 130 minutes (mean = 122.5 min) |
|  | Give examples of how iteration occurred during the process: | Card sorting technique (WS1) was repeated in WS6 to ensure informed decision-making and to gain information on participants awareness of the relevance of suggested LiSA contents for the early identification of health risks. The differences between the card sorting results from WS1 and WS6 were discussed in the group afterwards. |
| Conducting Frameworks | Which frameworks did you use? Why? | PRODUCES framework as a guideline to conduct the co-creation process [33,34] |
|  | Were there any frameworks or models you used that are not mentioned in this guideline? If yes, please list them: | - |
| **Evaluating** | | |
| Evaluating Methods | Which methods did you use? Why? | Qualitative content analysis [39] to systematically evaluate audio-recorded and written material.  Optimized Honeycomb Model for UX [40,41]: In the coding process, this model served as a basic structure to categorize components affecting the LiSA UX thematically. Data material was deductively sorted to the seven Honeycomb Model categories (findable, accessible, usable, desirable, credible, useful, and valuable). Further categories were inductively added, and subcategories were created within all categories. Data from flipcharts, photos, and researchers’ notes were also viewed and additionally assigned to the categories.  Questionnaires: The affinity for technology interaction (ATI) scale [36] and the technology commitment short scale [37] were used to quantify participants’ experience with technology use. The short version of the User Experience Questionnaire (UEQ-S) [38] was used to measure participants’ subjective impression towards the UX of the tested apps. Quantitative data was analyzed descriptively. |
|  | Were there any methods you used that are not mentioned in this guideline? If yes, please list them: | - |
| Evaluating Frameworks | Which frameworks did you use? Why? | - |
|  | Were there any frameworks or models you used that are not mentioned in this guideline? If yes, please list them: | - |
| Process | Explain how co-creator satisfaction and contribution are evaluated (for example reporting on attendance rates, questionnaires, and interviews). | Due to vacation and illness, an average of eight people per workshop participated. One participant was excluded from the study after workshop 1 due to non-compliance with the workshop ground rules. A substitute participant was recruited, who then participated in WS3-6. Feedback on the workshops and suggestions for changes were asked for after each workshop. WS6 was concluded with participants sharing their feedback on the co-creation process overall. |
|  | How are results reported back to stakeholders and the public? | A German translation of the published results will be sent to all workshop participants. The final LiSA version will be available for the public use/ to the general public (e.g., via download in the App Store). |
| Outcome | Explain how the validity of the outcome and the process were evaluated (for example, face validation, and member checking): | During the workshops, methods for securing results (face validation and member checking [34]) were used. Each WS sharted with a short wrap-up of the last workshop and participants were invited to share their thoughts on the last workshop. |
|  | Explain plans for formal testing of the effectiveness/ scalability of the co-created outcome: | The next step is to develop a LiSA prototype building on the findings from the co-creation process. An initial prototype is to address users with few barriers. This prototype is then to be iteratively tested, further developed, and expanded in order to gradually overcome barriers and offer LiSA to a larger target group in the long term.  Other relevant stakeholders should be involved in future co-creation processes to discuss the LiSA concept: general practitioners, other HCPs (e.g., physiotherapists, optometrists), data privacy experts, health insurance companies and family members.  Further research steps will be the examination of the test quality criteria, i.e., test-retest reliability and cross-validation, in order to verify whether LiSA provides comparable data to a standard comprehensive geriatric assessment. |
|  | Explain the outcome of the evaluation (if tested): | - |
