# Supplementary material for: Co-Creating a Digital Life-Integrated Self-Assessment for Older Adults: User Experience Study
Source: JMIR Aging. 2023 Sep 26;6:e46738. doi: 10.2196/46738 (PMC10565622; doi:10.2196/46738)
Supplement: Multimedia Appendix 2 [file aging_v6i1e46738_app2.docx]

# Multimedia Appendix 2: Overview of the workshop contents.

| **no.** | **main topics** | **workshop agenda and methods** |
| --- | --- | --- |
| **1** | introduction, LiSA concept presentation,  participants’ initial thoughts about LiSA,  LiSA assessment contents | Introduction   - introduction round: participants, research team - LiSA concept presentation (Figure 1), example of a fall-risk self-assessment app: “Aachener Sturzpass” (fall risk prediction tool, Table 1, No. 1) - agenda (6 workshops) & co-creation roles - ***participants’ first thoughts about LiSA   LiSA assessment contents   - card sorting by two groups: “which assessment domains should (not) represented in LiSA” - ***differences/similarities between the group results   Homework   - all participants: test the “starthilfe digital” app (digital starter kit, Table 1, No. 2): introduction to basic tablet functions - 3 volunteers to use a step counting: document daily steps count for one week - fill out the affinity for technology interaction (ATI) scale |
| **2** | assessment domain: physical activity, physical function | - short recap on LiSA concept, questions & answers on previous WS - ***sharing experiences with the “starthilfe digital” app (digital starter kit), *fill out the UEQ, ***which components should (not) be adopted for LiSA”   physical activity   - short presentation (CPJ): physical activity assessment - ***discuss participants’ step counting data - ***“how should physical activity be assessed within LiSA”   strength and balance/physical function   - short presentation (CPJ): strength and balance assessment - test the “Up&Go” app (instrumented Timed-Up and Go Test, Table 1, No. 3): 2 participants volunteered as test persons, others observed, *fill out UEQ, ***sharing experiences from tester and observer point of view, “could this app be a part of LiSA?” - **test the Chair Rise test (1 tester, 1 observer, changing roles), ***sharing experiences from tester and observer point of view, “could this test be realized as a digital self-assessment in LiSA?”   Homework   - test the “Bewegungspass” (mobility passport, fitness self-assessment and training brochure, Table 1, No., 4) - fill out the technology commitment scale |
| **3** | assessment domain: cognition, physical activity | - questions & answers on previous WS   cognition   - short presentation (CM): cognition assessment - **test the “TUCAN” (TUebingen Cognitive Assessment for Neuropsyciatric disorders, Table 1, No. 5): 1 tester, 1 observer, changing roles, *fill out the UEQ, ***sharing experiences from tester and observer point of view, “could this app be a part of LiSA?”   physical activity   - ***sharing experiences with the homework: “Bewegungspass” (mobility passport , fitness self-assessment and training brochure), “could this be digitalized and used as a part of LiSA?”   Homework   - fill out the “LUCAS Navigator” questionnaire (self-test of functional competence, Table 1, No. 6) |
| **4** | assessment domain: vision, hearing | - questions & answers on previous WS   vision   - short presentation (optician expert): „vision assessment“ - *test the “ZEISS Online Vision Screening” (Table 1, No. 7), *fill out the UEQ, ***sharing experiences, “could this app be a part of LiSA?”   hearing   - short presentation (CB, MB): „hearing assessment“ - *test the “Mimi Hearing Test” app (Table 1, No. 8), *fill out the UEQ, ***sharing experiences, “could this app be a part of LiSA?” |
| **5** | assessment domain: social and contextual factors, outlook LiSA follow-up | - questions & answers on previous WS   social and contextual factors   - short presentation (CB, MB): „social and contextual factors“ - ***“should social and contextual factors be a part of LiSA?”   outlook on possible LiSA-follow-up interventions   - presentation of the two apps (NM, KGO) that could be used in case risks are identified by LiSA   - “smartVERNETZT” (German version of PRISM, app to reduce social isolation and loneliness in older adults, Table 1, No. 9)   - “KOKU” (German version, home training app for older adults to improve muscle strength, Table 1, No. 10): - *fill out the UEQ for smartVERNETZT and KOKU, ***questions & answers, “what do you think about smartVERNETZT and KOKU?” |
| **6** | LiSA assessment contents, user types, benefits & risks, end of workshop series | - questions & answers on previous WS   LiSA assessment contents   - repetition of card sorting: two groups (different group constellation compared to WS1): “which assessment domains should/shouln’t be represented in LiSA” - ***“differences between card sorting results WS1 and WS2   user types   - **personas (representing older adults with different levels of competence and motivation regarding technology use): get familiar with one persona, make notes: would this persona be a LiSA user/non-user, reasons for using /not using - ***each tandem presents their results, “are there other (non)-user types regarding LiSA/for whom should LiSA be developed”   LiSA benefits & risks   - ***questions & answers about data security (LR), pros/cons for using LiSA, what benefits/chances or disadvantages/risks do participants expect from using LiSA   end of workshop series   - summary about workshops (MB) - ***feedback from participants: “what did you like/dislike”, take-home message |

*Notes.* *individual work, **tandem work, ***in plenary/group discussion, UEQ = the User Experience Questionnaire, WS = workshop
