# Supplementary material for: Co-Creating a Digital Life-Integrated Self-Assessment for Older Adults: User Experience Study
Source: JMIR Aging. 2023 Sep 26;6:e46738. doi: 10.2196/46738 (PMC10565622; doi:10.2196/46738)
Supplement: Multimedia Appendix 3 [file aging_v6i1e46738_app3.docx]

# Multimedia Appendix 3: Description of personas, results from the tandem work, strategies to overcome potential user barriers.

| **description of personas (representing older adults with different attitudes towards technology and health)** | | | | |
| --- | --- | --- | --- | --- |
| Name (age) | **Sylvia (78)** | **Klaus (80)** | **Herbert (76)** | **Anita (70)** |
| ICT use | tablet, smartphone, computer | smartphone, computer | smartphone, computer, wife uses a tablet | cell phone (no internet access) |
| activity level | nordic walking, swimming, biking | goes shopping by foot | biking | gardening on the balcony |
| comorbidities | vision impairment (glasses), osteoporosis | overweight, hearing impairment (hearing aids) | diabetes, experienced a fall last year, knee pain | experienced a fall last year, overweight, post hip replacement surgery |
| social contacts | husband, children, grandchildren, nordic walking group, neighbors | wife, no children, neighbors | wife, children, grandchildren, friends | sister living in the neighborhood |
| attitude towards technology | - good knowledge in the use of various technical devices - would like to stay up to date with technology - sometimes needs family support | - likes to be well-connected - wants to stay up to date with technology | - basic knowledge in using smartphone and computer - skeptical about data protection - avoids technology, but knows that he can’t "escape" it entirely | - does not want to have anything to do with technology - TV is enough for her - fears dangers from the internet |
| attitude towards health | - "You're only as old as you feel." - regular health checks are important for her - so far, she has always taken good care of her health | - enjoys retirement - knows that physical activity is important, but prefers staying at home - would like to do more for his health | - health is an annoying topic, but important - realizes that his health isn’t the same as it used to be - wants to stay fit | - feels that her body no longer copes with anything - feels helpless regarding her health - goes to the doctor often, but her health doesn’t improve |
| **results from the tandem work** | | | | |
| Would this persona be a user/non-user? | user | user | user/non-user | non-user |
| Why would this persona be a user (+)  or non-user (-) ? | - interested in technology (+) - motivated to take care of her health (+) - already owns and uses tablet (+) - supported by the family (+) | - interested in technology (+) - has some experience in technology use (+) - motivated to do more for his health (+) | - has some experience with using technology, wife has a tablet (+) - family could possibly support him (+) - wants to avoid technology (-) - wants to care about his health (+) | - no experience in the use of smartphones or tablets (-) - not interested in technology (-) - frustrated about health (-) - has no one to support her (-) |

## **Strategies to overcome user barriers**

**
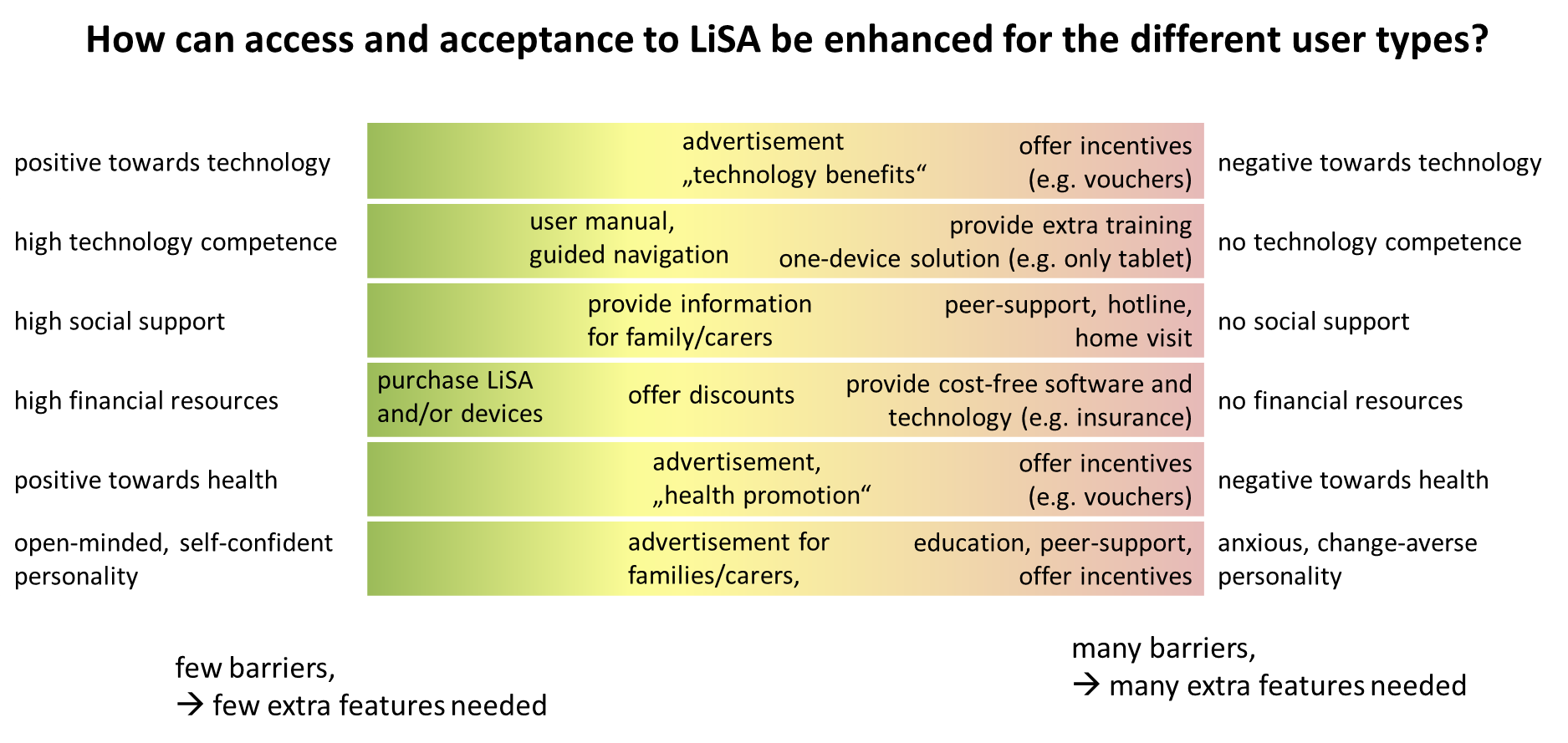
**
