# Supplementary material for: Co-Creating a Digital Life-Integrated Self-Assessment for Older Adults: User Experience Study
Source: JMIR Aging. 2023 Sep 26;6:e46738. doi: 10.2196/46738 (PMC10565622; doi:10.2196/46738)
Supplement: Multimedia Appendix 4 [file aging_v6i1e46738_app4.docx]

# Multimedia Appendix 4: Results from the Affinity for Technology Interaction and Technology Commitment Scale.

## Affinity for Technology Interaction (ATI) Scale (Franke, Attig & Wessel, 2019): participants’ scores

| response scale: completely disagree = 1, largely disagree = 2, slightly disagree = 3, slightly agree = 4, largely agree = 5, completely agree = 6 | | | | | | |
| --- | --- | --- | --- | --- | --- | --- |
| **item** |  | **Mean** | **SD** | **Min** | **Max** | **Range** |
| 1 | I like to occupy myself in greater detail with technical systems. | 3.56 | 1.59 | 1 | 6 | 5 |
| 2 | I like testing the functions of new technical systems. | 3.44 | 1.51 | 1 | 6 | 5 |
| 3 | I predominantly deal with technical systems because I have to.* | 3.00 | 1.22 | 1 | 5 | 4 |
| 4 | When I have a new technical system in front of me, I try it out intensively. | 3.22 | 1.20 | 2 | 5 | 3 |
| 5 | I enjoy spending time becoming acquainted with a new technical system. | 2.78 | 1.39 | 1 | 5 | 4 |
| 6 | It is enough for me that a technical system works; I don’t care how or why.* | 3.22 | 1.56 | 1 | 6 | 5 |
| 7 | I try to understand how a technical system exactly works. | 3.89 | 0.93 | 2 | 5 | 3 |
| 8 | It is enough for me to know the basic functions of a technical system.* | 2.33 | 1.22 | 1 | 5 | 4 |
| 9 | I try to make full use of the capabilities of a technical system. | 3.33 | 1.32 | 2 | 6 | 4 |
|  | **mean all items** | 3.20 |  |  |  |  |
|  | **SD** | 0.84 |  |  |  |  |
|  | **median** | 3.33 |  |  |  |  |
|  | **Cronbach's alpha** | 0.808 |  |  |  |  |

*Notes: *Responses to the three negatively worded items (items 3, 6, 8) were reversed (6=1, 5=2, 4=3, 3=4, 2=5, 1=6), SD = standard deviation*

## Technology Commitment Scale (Neyer, Felber & Gebhardt, 2016): participants’ scores

| response scale: completely disagree = 1, rather disagree = 2, partly agree = 3, rather agree = 4, completely agree = 5 | | | | | | |
| --- | --- | --- | --- | --- | --- | --- |
| **item** |  | **Mean** | **SD** | **Min** | **Max** | **Range** |
| 1 | I am very curious about new technical developments. | 3.33 | 0.87 | 2 | 5 | 3 |
| 2 | For me, dealing with technical innovations is mostly too much of a challenge.* | 3.22 | 0.44 | 3 | 4 | 1 |
| 3 | I find it difficult to deal with new technology - I simply can't do it most of the time.* | 3.33 | 0.71 | 2 | 4 | 2 |
| 4 | It's up to me whether I succeed in using new technical developments - it has little to do with chance or luck. | 3.67 | 0.87 | 2 | 5 | 3 |
| 5 | I am always interested in using the latest technical devices. | 2.33 | 1.12 | 1 | 4 | 3 |
| 6 | When dealing with modern technology, I am often afraid of failing.* | 3.56 | 1.24 | 2 | 5 | 3 |
| 7 | If I have difficulties in dealing with technology, it depends on me alone in the end to resolve them. | 3.00 | 0.87 | 2 | 4 | 2 |
| 8 | If I had the opportunity, I would use technical products much more often than I currently do. | 2.89 | 0.93 | 2 | 5 | 3 |
| 9 | I'm afraid of destroying new technical developments rather than using them properly.* | 3.67 | 1.00 | 2 | 5 | 3 |
| 10 | What happens when I engage with new technical developments is under my control in the end. | 3.11 | 0.93 | 2 | 4 | 2 |
| 11 | I quickly find myself enjoying new technical developments. | 3.00 | 1.22 | 1 | 5 | 4 |
| 12 | Whether I am successful in using modern technology depends largely on me. | 3.56 | 0.73 | 3 | 5 | 2 |
|  |  | **mean** | **SD** | **median** | **Cronbach's alpha** | |
|  | all items | 3.22 | 0.43 | 3.08 | 0.656 | |
|  | technology acceptance (item 1, 5, 8, 11) | 2.89 | 0.89 | 2.75 |  | |
|  | technology competence (item 2, 3, 6, 9) | 3.44 | 0.74 | 3.25 |  | |
|  | technology control convictions (item 4, 7, 10, 12) | 3.33 | 0.55 | 3.25 |  | |

*Notes: original items and response scale are in German and were translated for this article,
*Responses to the negatively worded items (items 2, 3, 6, 9) were reversed (5=1, 4=2, 2=4, 1=5), SD = standard deviation*
