# Supplementary material for: Co-Creating a Digital Life-Integrated Self-Assessment for Older Adults: User Experience Study
Source: JMIR Aging. 2023 Sep 26;6:e46738. doi: 10.2196/46738 (PMC10565622; doi:10.2196/46738)
Supplement: Multimedia Appendix 5 [file aging_v6i1e46738_app5.docx]

# Multimedia Appendix 5: Participants’ ratings of the tested apps on the short version of the User Experience Questionnaire, and dos and don’ts regarding the Life-integrated Self-Assessment.

## Short version of the User Experience Questionnaire (UEQ-S, Schrepp et al., 2017): ratings for the apps tested within the workshops

|  |  | **pragmatic quality** | | | **hedonic quality** | | | **overall evaluation** | | |
| --- | --- | --- | --- | --- | --- | --- | --- | --- | --- | --- |
|  | available data (n) | mean* | SD | comparison to benchmark** | mean* | SD | comparison to benchmark** | mean* | SD | comparison to benchmark** |
| tablet/ smartphone app: “starthilfe digital” | 8 | 1.81 | 0.74 | excellent | 1.34 | 1.11 | good | 1.58 | 0.64 | good |
| smartphone app: “Up&Go” | 7 | 1.36 | 1.18 | above average | 1.32 | 1.60 | good | 1.34 | 1.23 | good |
| tablet app: “TUCAN” | 9 | 1.80 | 0.85 | excellent | 1.11 | 1.70 | above average | 1.44 | 1.19 | good |
| web-based app: “ZEISS Online Vision Screening” | 7 | 2.00 | 0.75 | excellent | 1.04 | 1.31 | above average | 1.52 | 1.00 | good |
| tablet/smartphone app: “Mimi Hearing Test” | 7 | 0.25 | 1.59 | bad | 1.04 | 1.60 | above average | 0.64 | 1.55 | below average |
| tablet app: “smartVERNETZT” | 6 | 1.92 | 1.00 | excellent | 1.75 | 0.95 | excellent | 1.83 | 0.81 | excellent |
| tablet app: “KOKU” | 6 | 2.21 | 0.49 | excellent | 1.33 | 0.77 | good | 1.77 | 0.56 | excellent |

***Notes:*** *SD = standard deviation, * > 0.8 = positive evaluation (green), - 0.8 - 0.8 = neutral evaluation (yellow), < - 0.8 negative evaluation, **comparison to benchmark: excellent = in the range of the 10% best results, above average = 25% of results are better and 50% of results are worse, good = 10% of results are better and 75% of results are worse, below average = 50% of results are better and 25% of results are worse, bad = in the range of the 25% worst results*

## Dos and don’ts regarding LiSA derived from participants’ feedback on the tested apps

| **tested app, “name”** | **derived dos and don’ts regarding LiSA ( 🡪 category to which topics were assigned in the qualitative content analysis)** |
| --- | --- |
| tablet/ smartphone app:  “starthilfe digital“ (digital starter kit) | Dos   - possibility to use program with multiple devices 🡪 *useful/* *interoperability with other devices* - instructional video 🡪 *usable/instruction and exercise options, useful/auditive and visual elements* - virtual character and target group ambassadors guiding through the program 🡪 *desirable/program guide* - senior-oriented form of address 🡪 *desirable/program guide* - possibility to continue where you left off 🡪 *findable/navigation*   Don’ts   - senior-oriented form of address 🡪 *desirable/program guide* |
| smartphone app: “Up&Go” (instrumented Timed Up and Go test) | Dos   - voice instructions 🡪 *useful/ auditive and visual elements* - result displayed using a traffic light 🡪 *useful/displayed result/recommendation for action* - large font, not too much text 🡪 *usable/readable and visible* |
| tablet app: “TUCAN” (Tuebingen cognitive assessment for neuropsychiatric disorders) | Dos:   - exciting tasks 🡪 *desirable/fun* - recognizable goal behind the task 🡪 *credible/measurement accuracy*   Don’ts   - no buttons to navigate back and forth 🡪 *findable/navigation* - small font and illustrations 🡪 *usable/readability and visibility* - too many repetitions are boring 🡪 *desirable/fun* |
| web-based app: “ZEISS Online Vision Screening” | Dos:   - procedure similar to a standard visual assessment with an optician 🡪 *credible/measurement accuracy* - contacts of the optical providers in the neighborhood are displayed upon receiving the result 🡪 *useful/contact mediation*   Don’ts   - tasks not recognizable due to light reflection on the screen 🡪 *usable/readability and visibility* - assessments that have already been completed are displayed 🡪 *findable/easy and guided navigation* - time of day/lighting conditions not considered 🡪 *credible/measurement accuracy* - test did not stop when difficulty level was too high, user is forced to guess 🡪 *useful/adequate level of difficulty* |
| tablet/smartphone app: “Mimi Hearing Test” | Dos:   - exercise round before the assessment start 🡪 *usable/instruction and exercise options* - test procedure similar to a standard hearing assessment with otologist 🡪 *credible/measurement accuracy*   Don’ts:   - buttons disappearing too quickly, difficult navigation 🡪 *findable/easy and guided navigation* - test performed in a noisy environment 🡪 *usable*/*appropriate* *ambient conditions* |
| tablet app: “smartVERNETZT” (PRISM, Personal Reminder Information and Social Management) | Dos:   - provide Information and suggestions on what to do or change 🡪 *useful/displayed result and recommendation for action, valuable/change health behaviour* - clear app and menu navigation 🡪 *findable/easy and guided navigation* - tutorial videos and examples on how to use the app 🡪 *usable/instruction and exercise options* |
| tablet app: “KOKU” (Keep On Keep Up, home-based strength and balance exercise) | Dos:   - virtual person (sympathetic and funny) guiding through the program 🡪 *desirable/program guide, desirable/fun* - displayed result 🡪 *useful/displayed result and recommendation for action* - monitoring of results 🡪 *valuable/* *improve health-related self-efficacy* - rewarding elements (e.g. phrases) 🡪 *desirable/rewards and incentives* - various exercises with increasing difficulty 🡪 *useful/adequate level of difficulty* - reminders for the app use 🡪 *useful/reminders* |
